# Pediatric use of prescribed melatonin in Sweden 2006–2017: a register based study

**DOI:** 10.1007/s00787-020-01598-1

**Published:** 2020-07-22

**Authors:** Elin E. Kimland, Carola Bardage, Julius Collin, Anders Järleborg, Rickard Ljung, Anastasia N. Iliadou

**Affiliations:** 1 Swedish Medical Products Agency, Dag Hammarskjölds väg 42, Box 26, 751 03 Uppsala, Sweden; 2 National Board of Health and Welfare, Rålambsvägen 3, 116 30 Stockholm, Sweden

**Keywords:** Child, Adolescents, Melatonin, Prescription, Drug registry, ADHD, Sleep disturbances, Long-term

## Abstract

Sleep disturbances are common in the pediatric population and should primarily be treated non-pharmacologically. Most medicines for sleep disturbances are not approved for pediatric use and data on long-term safety is scarce. In Sweden, melatonin is classified as a prescription medicine. The aim of the present study was to characterize the prevalence and incidence of dispensed melatonin prescriptions, long-term treatment, concomitant dispensation of psychotropic medication, and psychiatric comorbidity, in children and adolescents aged 0–17 years living in Sweden during 2006–2017. Data was retrieved by linking the national population-based registers, the Swedish Prescribed Drug register and the National Patient register. In 2017, nearly 2% of the pediatric population 0–17 years was dispensed at least one prescription of melatonin, which was more than a 15-fold increase for girls and a 20-fold increase for boys, when compared to 2006. Among the children in the age group 5–9 who initiated a melatonin treatment in 2009, 15% of girls and 17% of boys were found to be continuously prescribed melatonin 8 years later. Nearly 80% of all children with dispensed melatonin had concomitant dispensations of psychotropic medications. The most common combination was melatonin together with centrally acting sympathomimetic medicines (23% of girls and 43% of boys). About half of the children (47% of girls and 50% of boys) had at least one registered diagnosis of mental or behavioral disorders. The most common diagnosis was attention deficit hyperactive disorder, across all age groups and genders. The continuous increase of use of melatonin in children, often concomitant with other psychotropic medications, together with a high proportion of younger children with prescriptions of melatonin on a long-term basis, suggests the need for further structured follow up studies, in particular of long-term use.

## Introduction

Pediatric sleep disturbances, especially insomnia, are common among children [1, 2], and even more frequent among children and adolescents with a psychiatric comorbidity [3]. Sleep disorders among children and adolescents can have a significant impact on daytime functioning and development, including learning, growth, behaviour, and emotion regulation, and treatment is therefore important [1, 4].

International and national guidelines on treatment of sleeping disorders recommend that children’s sleep disorders should primarily be treated with non-pharmacological interventions, such as structural sleeping habits. Medicines should only be used for a short period of time and only after careful consideration [5–8]. Several medicines are authorized based on clinical studies in adults, rendering insufficient knowledge about efficacy and safety for use in the pediatric population [9]. Hence, most medicines that are usually used for sleep disturbances in adults are not approved for children, and consequently, if used, are prescribed off-label [10, 11]. Melatonin is the most commonly used medicine for sleep disturbances among children and adolescents [11–15].

Melatonin is a hormone, secreted primarily by the pineal gland at night under normal light–dark conditions [16]. Possible physiological effects of melatonin are various, and include detoxification of free radicals and antioxidant actions, bone formation and protection, reproduction, cardiovascular, immune or body mass regulation [16]. Authorization status for melatonin varies among countries. Melatonin is available as an over the counter drug in some European countries, while in the USA it is regarded as a dietary supplement. In Sweden and most European countries, melatonin is available as a prescribed medicine approved for use in adults over 55 years of age, since 2007. Prior to 2007, melatonin was prescribed on a license as an unauthorized medicine. In Sweden, all physicians can prescribe melatonin to children, either off-label or on a license. At the time of the study, no medicine containing melatonin was approved for the pediatric population.

Among the Nordic European countries there has been an increase in prescriptions of melatonin among children and adolescents [11–13]. Studies among children and adolescents with attention deficit hyperactive disorder (ADHD) and other neurodevelopmental disorders indicate that melatonin can be effective in treating sleep disorders in these patient groups [6–8, 15]. Pre-clinical studies using experimental animals have shown potential effects on the reproductive system, which appears to be influenced by dosage and dosage-timing [17–19]. However, the relevance of these findings for safety in humans is unknown. Clinical trials and some human studies have shown that adverse events following short-term treatment were generally minor, transient and easily managed, and mostly related to fatigue, mood, or psychomotor and neurocognitive performance [17]. However, little is known regarding safety and adverse effects of long-term use in the pediatric population [17, 20, 21]. Considering the multiple physiological effects of melatonin and the scarce knowledge of safety, along with an exponential rise in use, it is of interest for health care professionals, care givers and medical regulatory authorities to investigate melatonin use among the pediatric population.

The aim of this register-based study was to characterize the prevalence and incidence of dispensed melatonin prescriptions, long-term treatment, concomitant dispensation of psychotropic medication, and psychiatric comorbidity, according to age and gender in children and adolescents aged 0–17 years living in Sweden, during 2006–2017.

## Method

### Study population

The study population was comprised of children between 0 and less than 18 years of age (0–17 years) living in Sweden, between the years 2006 (number of children 1,934,080) and 2017 (number of children 2,099,005).

### National registers

Data were retrieved by linking national population-based registers based on the personal identity number [22] assigned to each Swedish resident at birth or immigration to Sweden. The national registers used were the Swedish Prescribed Drug register [23] and the National Patient register [24].

The *Swedish Prescribed Drug register,* held at the National Board of Health and Welfare, records data from July 2005 and contains all community pharmacy dispensed and prescribed medicines classified according to the *Anatomic Therapeutic Chemical Classification system* (*ATC*) [25]. The drug register has almost complete coverage (missingness < 1%) [23]. From the register, information was obtained on dispensed medicines, dates of dispensation, and dosage text written in the prescription by the physician.

The *National Patient register*, held at the National Board of Health and Welfare, records data from 1964 and contains information on all reported cases of inpatient care and doctor visits in the specialized outpatient care. The inpatient part of the register has full coverage from 1987. Information from the outpatient part was included in the register from 2001 and gained an increased coverage over the subsequent years [24]. The register contains both main and several contributory diagnoses. Since 1997, diagnoses are classified according to the Swedish version of the International Statistical Classification of Diseases and Related Health Problems—Tenth Revision (ICD 10) [26]. The validity of the register is high with a positive predictive value of 85–95% for most of the diagnoses [24]. Data from the primary care, as well as data from the specialized outpatient care by occupational groups, other than doctors, is not available in the National Patient register.

## Analysis of dispensed melatonin prescriptions

### Prevalence and incidence

The prevalence of dispensed melatonin (ATC code-N05CH01) per calendar year was defined as the number of children with at least one dispensed prescription for melatonin during that calendar year per 1000 children in the population. The incidence (new users) of melatonin per calendar year was defined as at least one dispensed prescription for melatonin during that calendar year without any dispensed prescription for melatonin within the previous two calendar years, per 1000 children in the population. Prevalence and incidences are presented by gender and age group (0–4, 5–9, 10–12 and 13–17 years of age). Median age per calendar year in the whole age group 0–17 years of age is also presented.

### Average prescribed dose

In order to estimate the average daily melatonin dose, a random sample of 100 prescriptions per gender and age group dispensed during 2016 was selected, in total 800 prescriptions. One of the authors (EK) manually examined the 800 dose texts and calculated the prescribed daily dose for each prescription based on the specified dose in each recipe. Data is presented as averages with standard deviation, median and interquartile range by age group.

### Long-term treatment

Children 0–17 years of age and classified as new melatonin users in 2009, without any prescriptions of melatonin 2007–2008 (two-year wash-out period), were followed until 2017 in order to estimate long-term treatment. The age groups are based on the age at the time of the first prescription. All children were followed for a total of 8 years. For example, a 17-year-old in 2009 was followed for subsequent melatonin prescriptions until the age of 25 (in 2017). Long-term treatment was defined as a continuous melatonin treatment including at least three dispensations of melatonin every subsequent calendar year after 2009. One dispensation usually covers medication for 3 months. Therefore, three or more dispensations during a calendar year was defined as long-term treatment. Subsequently, children with less than three dispensations of melatonin in any year were no longer regarded as being on long-term treatment. Children aged 0–4 years were few, and therefore the results from this age group should be interpreted with caution.

### Concomitant dispensation of psychotropic medication

Concomitant dispensation of psychotropic medications other than melatonin was defined as any drug prescription occurring during the same calendar year as the dispensation of the prescribed melatonin. The following psychotropic medications were studied; antidepressants (ATC code-N06A), anxiolytics (ATC code-N05B and phenothiazine derivatives ATC code-R06AD), antipsychotics (ATC code-N05A), antiepileptics (ATC code-N03A), hypnotics and sedatives excluding melatonin (ATC code-N05C) and centrally acting sympathomimetics (ATC code-N06B). The analysis was restricted to data during year 2017, and for ages 5–17. Due to very small numbers, the age group 0–4 was omitted.

## Psychiatric comorbidity

Psychiatric comorbidity was classified according to chapter V in ICD 10, up to the 3–character level (i.e. F90 hyperkinetic disorders) [26]. All psychiatric diagnoses, documented up to 6 months prior dispensing at least one melatonin prescription in 2017, among children 5–17 years of age were retrieved. Each diagnosis was only calculated once, with some children obtaining several diagnoses. Due to very small numbers, the age group 0–4 was omitted.

Descriptive tables and statistics were done in SAS Enterprise Guide, and figures in MS Excel.

The study was approved by the Ethical Review Board at Uppsala, Sweden (ethical approval Dnr 2017/439).

## Results

In 2006, there were 1,934,080 children living in Sweden, of which 1996 were children with at least one dispensed prescription of melatonin during that year. The corresponding numbers for 2017 were 2,099,005 children, where 36,212 children had at least one dispensation of melatonin (Table 1).

**Table 1:** Descriptive statistics (number and proportions) of the study population of children 0–17 years living in Sweden 2006–2017 according to sex

Calendar year												
Total number and proportion	2006	2007	2008	2009	2010	2011	2012	2013	2014	2015	2016	2017
Girls												
*N*	941,745	941,030	938,415	935,621	934,142	933,308	934,982	942,754	956,276	973,209	994,509	1,017,169
Dispensed melatonin *N* (‰)	680 (0.7)	838 (0.9)	1234 (1.3)	1775 (1.9)	2356 (2.5)	3099 (3.3)	4054 (4.3)	4497 (4.8)	5848 (6.1)	7869 (8.1)	11,941 (12.0)	15,447 (15.2)
New users dispensed melatonin *N* (‰)	–	–	698 (0.7)	1069 (1.1)	1403 (1.5)	1852 (2.0)	2314 (2.5)	2315 (2.5)	3312 (3.5)	4507 (4.6)	6873 (6.9)	8027 (7.9)
Median age (years)	12	12	13	14	14	14	14	14	14	13	14	13
Boys												
*N*	992,335	991,757	989,832	987,347	985,954	985,843	988,683	997,548	1,012,606	1,031,973	1,056,235	1,081,836
Dispensed melatonin *N* (‰)	1316 (1.3)	1670 (1.7)	2409 (2.4)	3300 (3.3)	4350 (4.4)	5592 (5.7)	7042 (7.1)	7980 (8.0)	9513 (9.4)	11,852 (11.5)	16,263 (15.4)	20,765 (19.2)
New users dispensed melatonin *N* (‰)	–	–	1264 (1.3)	1747 (1.8)	2240 (2.3)	2754 (2.8)	3293 (3.3)	3407 (3.4)	4084 (4.0)	5334 (5.2)	7404 (7.0)	8839 (8.2)
Median age (years)	12	12	12	12	13	12	12	12	12	12	12	12

There were 680 (0.7‰) girls and 1316 (1.3‰) boys with at least one dispensation of melatonin in 2006 (Table 1). In 2017, there were 15,447 (15.2‰) girls and 20,765 (19.2‰) boys with at least one dispensation of melatonin (Table 1). The number of children aged 0–17 years with only one dispensation of melatonin was 36% in girls and 35% in boys in 2006 and 25% in girls and 26% in boys in 2017.

### Prevalence of dispensed melatonin

**Fig. 1:**
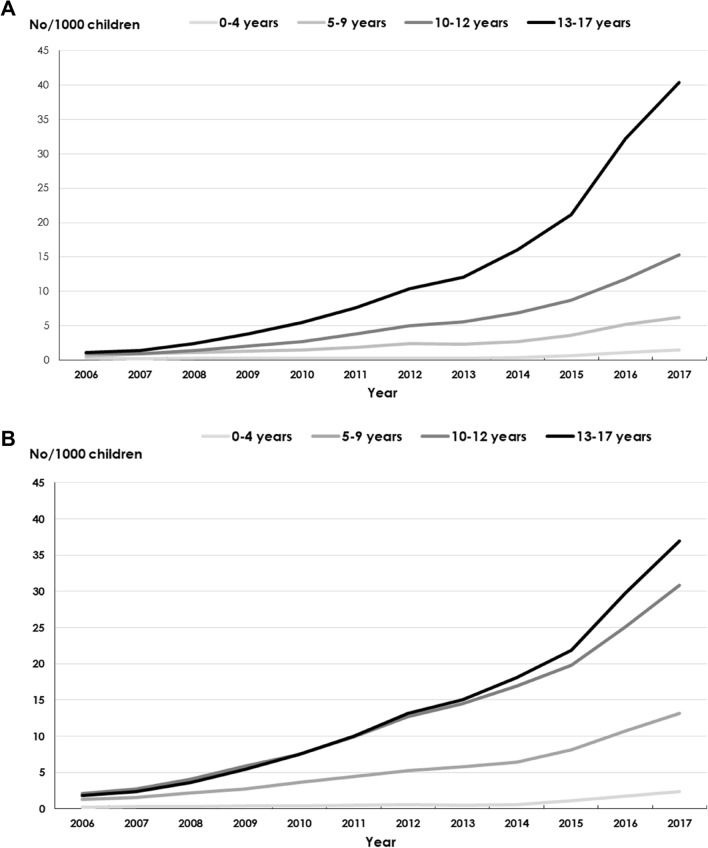
**a** Prevalence of dispensed melatonin prescriptions in girls per 1000, stratified by age groups, 2006–2017. **b** Prevalence of dispensed melatonin prescriptions in boys per 1000, stratified by age groups, 2006–2017

The prevalence of dispensed melatonin prescriptions among girls and boys (Table 1) in all age groups increased between 2006 and 2017 (Fig. 1a, b). During 2006, 0.7 girls per 1000 and 1.3 boys per 1000 were dispensed melatonin, compared to 15 girls per 1000 and 20 boys per 1000 during 2017. Girls 13–17 years of age accounted for the largest increase (Fig. 1a) of 37 times from 2006 to 2017 followed by boys 13–17 years of age (Fig. 1b) of 21 times per 1000 children. The corresponding number for girls and boys 10–12 years of age in 2017 was 17 and 15 times per 1000 children, respectively. Children 0–4 years of age had the lowest prevalence albeit with an increase from 2006 to 2017. The median age of children being dispensed melatonin for girls was 12 years in 2006 and 13 years in 2017 and the median age for boys was 12 years both in 2006 and 2017 (Table 1).

### Incidence of dispensed melatonin

**Fig. 2:**
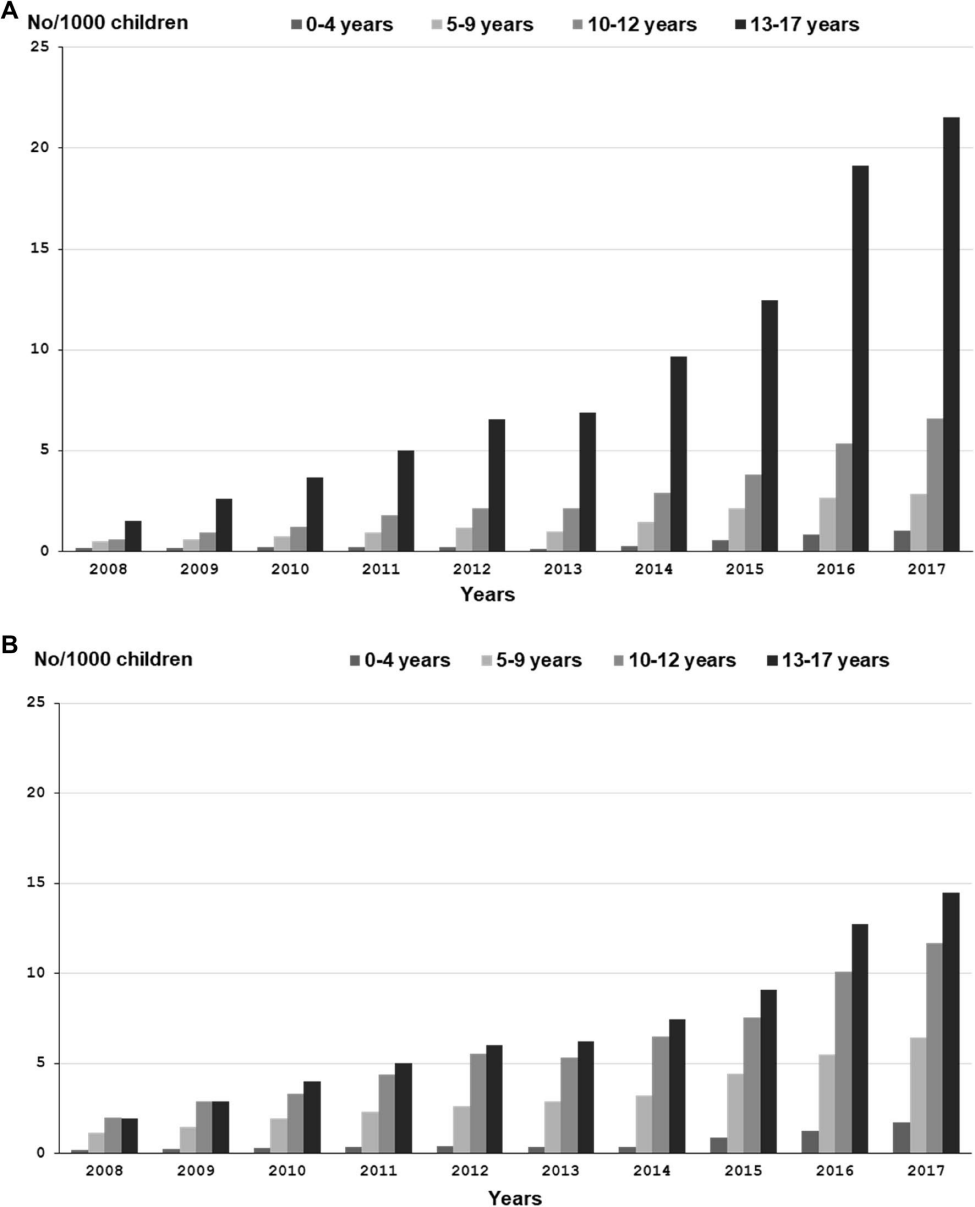
**a** Incidence of dispensed melatonin prescriptions in girls per 1000, stratified by age groups 2008–2017. **b** Incidence of dispensed melatonin prescriptions in boys per 1000, stratified by age groups 2008–2017

The incidence (new users) of melatonin increased in both girls and boys (Table 1) in all age groups (Fig. 2a, b) between 2008 and 2017. Adolescents aged 13–17 accounted for the highest increase (1.5–22 per 1000 for girls and 2.0–15 per 1000 for boys) followed by children aged 10–12 (0.6–6.6 per 1000 for girls and 2.0–12 per 1000 for boys). For children aged 5–9, the increase was 0.5–2.9 per 1000 for girls and 1.2–6.4 per 1000 for boys. The incidence among the youngest children aged 0-4 also increased from 0.2 per 1000 in girls and boys in 2006 to 1.0 per 1000 in girls and 1.7 per 1000 in boys in 2017.

### Average prescribed dose

The average prescribed dose was lowest (3.1 mg) for the youngest age group and highest (4.0 mg) for the oldest age group, with a similar range in all age groups (Table 2).

**Table 2:** Average (standard deviation), median dose (mg) of melatonin, and range in different age groups among children with at least one dispensed melatonin prescription in 2016

Age groups (years)	Number of prescriptions	Average (SD)	Median dose (mg)	Range (mg)
0–4	200	3.1 (1.7)	2	0.5–10
5–9	200	3.6 (2.0)	3	0.5–12
10–12	200	3.7 (2.0)^a^	4	1–12
13–17	200	4.0 (2.1)	4	0.5–15

^a^An outlier at 25 mg/daily was omitted from this table

### Long-term treatment

**Fig. 3:**
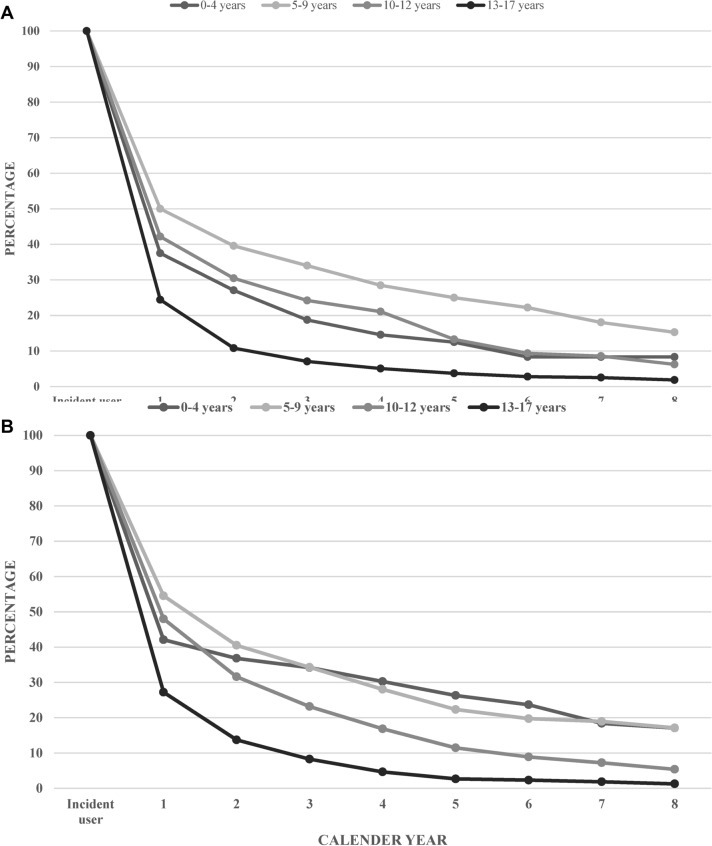
**a** Frequency of girls (new users) with continuous use of melatonin since 2009 followed until 2017. **b** Frequency of boys (new users) with continuous use of melatonin since 2009 followed until 2017

There were 2816 new users of melatonin (1069 girls and 1747 boys) among children 0–17 years in 2009. Children with at least three dispensations of melatonin 1 year after the first initiation in 2009, accounted for 36% of all new melatonin users. Five years after the first initiation of melatonin treatment in 2009, about 3% of children 13–17 years, girls and boys alike, were still dispensing at least three melatonin prescriptions per year (Fig. 3a, b). The corresponding figures for girls and boys aged 10–12 with at least three prescriptions per year were 13 and 12% after 5 years from initiation of treatment in 2009. The corresponding figures after 5 years, for girls and boys at the ages 5–9 years were 25 and 22%, respectively. The highest proportion of long-term melatonin dispensation after 5 years was observed among boys 0–4 years of age (26%) and girls 5–9 years of age (25%). However, the number of boys 0–4 years of age (*N* = 76) was small and the result should therefore be interpreted with caution. Eight years after the initial dispensation of melatonin, 5% of girls and 7% of boys aged 0–17 years, had at least three dispensations of melatonin within a year. The highest long term use of melatonin, after 8 years from first dispensation, was observed among children 5–9 years of age (15% girls and 17% boys).

### Concomitant dispensation of psychotropic medication

In 2017, most girls (11,444, 76%) and boys (15,652, 78%) between the ages 5–17 years were dispensed melatonin together with at least one concomitant psychotropic medication (Table 3). About 23% of girls and 43% of boys had a concomitant dispensation of another centrally acting sympathomimetic medicine, the majority attributable to medicines for ADHD (Table 3). Among girls, 9% had a concomitant dispensation of an antidepressant and nearly 7% a concomitant dispensation of an anxiolytic, compared to about 4% among boys with a concomitant dispensation of an antidepressant or an anxiolytic (Table 3). About 1% of children 5–17 years of age had a concomitant use of melatonin with a neuroleptic medicine or a medicine for epilepsy. Melatonin without a concomitant psychotropic medication was most commonly used in children aged 5–9. Melatonin together with a concomitant medication for ADHD was most common among boys and girls aged 10–12. A combination of melatonin and several psychotropic medications was more common among boys and girls aged 13–17. Similar patterns of concomitant dispensations of psychotropic medications were found for girls (1284, 75%) and boys (2498, 78%) in 2009.

**Table 3:** Number and frequency of the most concomitant use of psychotropic medication among children 5-17 years with at least one dispensation of melatonin by age groups and sex in 2017

Age group (years)	All	Girls				Boys			
	5-17	5-17	5-9	10-12	13-17	5-17	5-9	10-12	13-17
Number of children with at least one dispensed melatonin prescription	35,033	15,017	1826	2586	10,605	20,016	4100	5504	10,412

Psychotropic medication	*N* (%)	*N* (%)	*N* (%)	*N* (%)	*N* (%)	*N* (%)	*N* (%)	*N* (%)	*N* (%)
Melatonin and drugs for ADHD (ATC-N06B)	12,062 (34)	3375 (23)	481 (26)	1008 (39)	1886 (18)	8687 (43)	1660 (41)	2918 (53)	4109 (40)
Melatonin (ATC-N05CH01), no other psychotropic medication	7937 (23)	3573 (24)	795 (44)	718 (28)	2060 (19)	4364 (22)	1313 (32)	976 (18)	2075 (20)
Melatonin and drugs for depression (ATC-N06A)	2223 (6.3)	1365 (9.1)	11 (0.6)	87 (3.4)	1267 (12)	858 (4.3)	39 (1.0)	125 (2.3)	694 (6.7)
Melatonin and drugs for depression and anxiety	2163 (6.2)	1647 (11)	10 (0.5)	86 (3.3)	1551 (15)	516 (2.6)	15 (0.4)	78 (1.4)	423 (4.1)
Melatonin and drugs for anxiety (ATC-N05B, R06AD) and ADHD	1579 (4.5)	617 (4.1)	88 (4.8)	161 (6.2)	368 (3.5)	962 (4.8)	237 (5.8)	320 (5.8)	405 (3.9)
Melatonin and drugs for ADHD and depression	1560 (4.5)	634 (4.2)	14 (0.8)	75 (2.9)	545 (5.1)	926 (4.6)	48 (1.2)	219 (4.0)	659 (6.3)
Melatonin and drugs for anxiety	1703 (4.9)	989 (6.6)	159 (8.7)	134 (5.2)	696 (6.6)	714 (3.6)	215 (5.2)	153 (2.8)	346 (3.3)
Melatonin and drugs for ADHD, depression and anxiety	871 (2.5)	516 (3.4)	7 (0.4)	41 (1.6)	468 (4.4)	355 (1.8)	25 (0.6)	91 (1.7)	239 (2.3)
Melatonin, neuroleptics (N05A) and drugs for ADHD	428 (1.2)	88 (0.6)	20 (1.1)	29 (1.1)	39 (0.4)	340 (1.7)	72 (1.8)	120 (2.2)	148 (1.4)
Melatonin and drugs for epilepsy (ATC- N03A)	417 (1.2)	198 (1.3)	51 (2.8)	47 (1.8)	100 (0.9)	219 (1.1)	78 (1.9)	60 (1.1)	81 (0.8)
Melatonin and neuroleptics	226 (0.6)	70 (0.5)	9 (0.5)	11 (0.4)	50 (0.5)	156 (0.8)	37 (0.9)	37 (0.7)	82 (0.8)
Melatonin and other sleeping agents (ATC-N05C)	114 (0.3)	64 (0.4)	^a^	^a^	63 (0.6)	50 (0.2)	^a^	^a^	48 (0.5)
Melatonin and other combinations of psychotropic drugs^b^	3750 (11)	1881 (13)	187 (10)	230 (8.9)	1980 (19)	1869 (9.3)	385 (9.4)	497 (9.0)	1342 (13)

^a^Due legal reasons, numbers that are less than five individuals are not shown in the table

^b^Antidepressants (ATC code-N06A), anxiolytics/phenothiazine derivative (ATC code-N05B/R06AD), antipsychotics (ATC code-N05A), antiepileptics (ATC code-N03A), hypnotics and sedatives excluding melatonin (ATC code-N05C), and centrally acting sympathomimetics (ATC code-N06B)

### Psychiatric comorbidity

Approximately half of all children and adolescents 5–17 years of age, who were dispensed melatonin in 2017, had at least one diagnosis of a mental and behavioral disorder (47% of girls and 50% of boys) documented up to 6 months prior to the dispensation of a melatonin prescription, se Table 4. Similar proportions of reported psychiatric diagnosis were seen among different age groups. However, only about one-third of girls 5–9 years of age had a documented psychiatric diagnosis. Among children with a documented diagnosis, the most frequent ones were behavioral and emotional disorders from the chapter F90–F98 in ICD10 both in girls (17%) and in boys (30%), the majority attributed to diagnosis of hyperkinetic disorders (F90), documented for 3348 girls and 6758 boys. Disorders of psychological development (F80–F89) were documented among 5.2% of girls and 10% of boys, nearly all attributed to autism spectrum disorders (1079 girls and 2373 boys). It was more common with neurotic, stress-related and somatoform disorders (F40–F48) among girls (11%) compared to boys (3.5%), attributed mainly to diagnosis of anxiety (1613 girls and 524 in boys). More girls (9.3%) had also a diagnosis of mood (affective) disorders (F30–F39) compared to boys (3.1%), mostly attributable to diagnosis of depression documented for 1735 girls and for 649 boys. A higher proportion of boys had a documented diagnosis of ADHD compared to girls across all age groups. A higher proportion of boys had also a documented diagnosis of autism compared to girls, across all age groups. A higher proportion of girls in the age group 13–17 had a documented diagnosis of depression compared to boys (Table 4). Similar patterns of psychiatric comorbidity were found for girls (42%) and boys (45%) in 2009.

**Table 4:** Number and frequency of the most frequent main psychiatric diagnosis among children 5–17 years with at least one dispensation of melatonin by age groups and sex in 2017

Age group (years)		All	Girls				Boys			
		5–17	5–17	5–9	10–12	13–17	5–17	5–9	10–12	13–17
Diagnosis	ICD 10 -code	*N* (%)	*N* (%)	*N* (%)	*N* (%)	*N* (%)	*N* (%)	*N* (%)	*N* (%)	*N* (%)
At least one psychiatric diagnosis	F chapter	22,469 (49)	10,352 (47)	800 (30)	1434 (44)	8118 (50)	12,117 (50)	2315 (41)	3332 (52)	6470 (54)
Mood (affective) disorders	F30–F39	2767 (6.0)	2014 (9.1)	^a^	54 (1.7)	1960 (12)	753 (3.1)	11 (0.2)	67 (1.0)	675 (5.6)
Depression	F32	2384 (5.1)	1735 (7.8)	^a^	51 (1.6)	1683 (10)	649 (2.7)	7 (0.1)	62 (1.0)	580 (4.8)
Neurotic, stress-related and somatoform disorders	F40–F48	3198 (6.9)	2342 (11)	26 (1.0)	168 (5.1)	2148 (13)	856 (3.5)	51 (0.9)	158 (2.5)	647 (5.4)
Anxiety	F41	2137 (4.6)	1613 (7.3)	15 (0.6)	105 (3.2)	1493 (9.2	524 (2.2)	20 (0.4)	109 (1.7)	395 (3.3)
Disorders of psychological development	F80–F89	3667 (7.9)	1161 (5.2)	197 (7.3)	225 (6.9)	739 (4.6)	2506 (10)	644 (11)	630 (9.8)	1232 (10)
Autism spectrum disorders	F84	3452 (7.5)	1079 (4.9)	173 (6.4)	213 (6.5)	693 (4.3)	2373 (9.8)	601 (11)	605 (9.4)	1167 (10)
Behavioral and emotional disorders	F90–F98	11,036 (24)	3731 (17)	461 (17)	881 (27)	2389 (15)	7305 (30)	1476 (26)	2353 (37)	3476 (29)
Hyperkinetic disorders	F90	10,106 (22)	3348 (15)	389 (14)	796 (24)	2163 (13)	6758 (28)	1311 (23)	2182 (34)	3265 (27)

^a^Due legal reasons number that are less than five individuals are not shown in this table

## Discussion

This nationwide study showed an exponentially increased dispensation of melatonin in children 0–17 years of age from 2006 to 2017 in Sweden. The increase in both prevalence and incidence of melatonin users was most noticeable in teenage girls and boys. Prescribed doses reported in this study correspond to recommended daily doses among adults. Nearly one-fifth of children aged 5–9 years being treated with melatonin, were continuously dispensed melatonin up to 8 years after their first prescription. More than three out of four of those with a melatonin treatment had a concomitant psychotropic medication, most commonly related to a hyperkinetic disorder. Similarly, around half of the children had a recent recording of psychiatric disorders, foremost related to hyperkinetic disorders.

The increasing prevalence and incidence of dispensation of melatonin prescriptions in children in our study has previously been shown in other Nordic European countries [11–13, 27]. A Norwegian study showed an increase in prevalence for girls 1.5–7.7 per 1000 and boys 3.4–11.0 per 1000 during the time period 2004–2012 [11]. The prevalence observed in the current study corresponds to the levels seen in 2012 in the Norwegian study, however, our study showed that the noted increase in incidence continued until 2017. Similarly, a Danish study showed that the number of melatonin prescriptions (all off-label) increased expansively from 2006 to 2012 [13]. Another Swedish study investigating melatonin use among children and adolescents 0–19 years of age between 2006 and 2013 reported similar results [12]. Furthermore, the Swedish study stressed the importance of continuous follow-up of melatonin use in a pediatric population. In the current study, boys were more often medicated from 10 years of age compared to girls who seemed to be medicated more often after 13 years of age. Similar findings have been shown in previous studies [11–13]. This is in line with studies suggesting that boys with ADHD are prone to receive medical treatment at an earlier age than girls [28]. If the increased prevalence and incidence of melatonin dispensation reflects an unmet need for medical treatment of increased sleeping disorders among children and adolescents, or perhaps an overtreatment, is not possible to answer based on the outcome of this study.

The present study showed that the daily dose dispensed to children and adolescents 0–17 years was slightly increasing with rising age. In line with our findings, a Norwegian study reported a median dose per day of 3 mg in boys and 2.5 mg in girls [11]. In a recent clinical trial on melatonin use compared to placebo, a dose between 3–5 mg daily was used in a pediatric population, similar to our presented results of an average prescribed daily dose in Swedish children [21].

Melatonin is foremost recommended to use as a short-term treatment for sleeping disorders. Based on the current results about two-thirds of all children were treated with melatonin on a short time basis. However, some children were treated for several years, which was also shown in a Norwegian study [29]. An interesting finding in our study was that young children at the ages 5–9 years was the age-group with the highest percentage of dispensed melatonin on a long-term basis (up to 5 years) compared to older children and adolescents. Boys aged 0–4 had similar long-term consumption of melatonin as boys 5–9 years of age. Girls aged 0–4 had similar 5-year continuous dispensation of melatonin as girls 10–12 years of age. However, the number of children aged 0–4 years of age were few, consequently data should be interpreted with caution for this age group.

The results indicate that fewer children in the older age groups are treated with melatonin for a long period of time. This might reflect a discontinuation of the need of medical treatment for these children for their sleeping disorders.

A majority of children in the age group 13–17 had reached adulthood during the study time period and a more likely explanation for the observed lower percentage of melatonin dispensations could be that other sleeping agents, such as benzodiazepines, were used instead [30]. However, since continuous dispensations of other sleeping agents apart from melatonin was not included in this study, it is unknown whether melatonin was replaced by other sleeping agents. A possible explanation for the lower proportion of long-term melatonin users in the age group 10–12 years could be the initiation of treatment for ADHD that leads to less sleeping disturbances and render in less need for long-term melatonin treatment.

Whether long term use of melatonin will prevail for children with a first dispensation in later years is difficult to foresee, since data are not available for 5 or 8 years ahead. However, the continuous increase in prevalence and incidence perhaps warrants the importance of follow up studies in this area.

Although children 5–9 years of age are treated for longer time periods, they do not have higher concomitant use of psychiatric medicines nor higher frequency of psychiatric diagnosis, which could suggest that the longer use of melatonin is not necessarily due to multiple psychiatric comorbidity.

Little is known regarding safety and adverse effects of long-term melatonin use. A systematic review of clinical evidence on orally administered melatonin showed that generally adverse events were transient and associated with day-time dosing [17]. The most frequently reported adverse events related to reductions in psychomotor and neurocognitive function or fatigue and excessive sleepiness, which are more related to the properties of melatonin per se [17, 18, 31]. It has been suggested that melatonin is involved in reproduction and sexual maturation [16, 20]. Our findings show that many children are treated with exogenous melatonin prior to and during puberty. It has been discussed whether long-term treatment with melatonin can influence sexual maturation among children. Human studies investigating the contraceptive properties of melatonin, administered 7.5–300 mg melatonin daily to healthy women and showed suppressed luteinizing hormone and subsequent ovulation within one menstrual cycle [32, 33]. Administration of 3 mg daily in men showed reduction in sperm concentration and motility, alongside with a reduction in estrogens and an increase in the androgen:estrogen ratio. However, this effect was only seen in a minority of the participants, in a very small study [33]. On the contrary, other studies showed protective effects on sperm parameters [34]. In two small pediatric studies (about 40 participating in each study) conflicting results were found regarding possible effect on pubertal development after melatonin treatment [19, 31]. Although reports are reassuring regarding short term safety of melatonin use, there is still a need for further studies on potential long-term effects in pubertal development.

In the current study almost 80% of children had a psychiatric comorbidity of mental or behavioral disorders. Most of the diagnosis in boys were related to ADHD, while in girls it was in addition to ADHD also more often related to mood disorders. The majority of children also had concomitant medication suggesting that melatonin was predominantly prescribed as a comedication in relation to another psychotropic medication, especially treatment for ADHD, which is in accordance with published literature [3, 7, 13–15]. This is further supported by an increased prevalence of psychotropic medicine use documented in children in many European countries [35]. Another interesting finding was that adolescent 13–17 years of age were more often treated with several psychotropic medications together with melatonin, especially girls 13–17 years of age. It has been shown in previous studies that a considerable proportion of children and adolescents with ADHD received treatment for their ADHD in combination with other antipsychotics [36, 37]. Therefore, it is important to continue monitoring children and their pharmacological treatment for mental and behavioral health problems [38]. The remaining 20% of the children, that did not have any documented psychiatric diagnosis, were interesting, and was more common among children 5–9 years of age. We speculate that these children may had entered the health care system with sleeping disorders and were probably under investigation for behavioral problems but had not received any psychiatric diagnosis yet, or may have received melatonin for other conditions, such as diseases of the nervous system (i.e. cerebral palsy.)

Strengths of the study include the large sample size, the population based and nationwide prospective coverage of all prescriptions, since melatonin is not available over-the counter, and all diagnoses in inpatient care and specialized outpatient care using national registers of high validity. The nationwide register-based design reduces possible recall bias and selection bias.

However, there were some limitations. The results were based on data of dispensed prescriptions and hence we assumed adherence to the dispensed medicines. The current study did not capture diagnoses of psychiatric disorders registered in a primary care setting. Also, whether a non-pharmacological treatment had preceded the pharmacological treatment with melatonin was not possible to analyse, since these interventions are not always recorded as specific diagnosis or procedures.

## Conclusion

In conclusion, the overall increase in use of melatonin among girls and boys in all ages, often concomitant with other psychotropic medications, and the high proportion of young children, who were prescribed melatonin on a long-term basis, suggests further need for structured follow up. Specifically, studies on long-term safety of melatonin use in children and adolescents are of importance.
